# Increased active pulmonary tuberculosis risk from sharing bong of cannabis: a case–control study from Thailand

**DOI:** 10.3389/fpubh.2024.1474761

**Published:** 2024-11-13

**Authors:** Kemmapon Chumchuen, Virasakdi Chongsuvivatwong

**Affiliations:** Department of Epidemiology, Faculty of Medicine, Prince of Songkla University, Songkhla, Thailand

**Keywords:** tuberculosis, cannabis, case–control, increased risk, bong

## Abstract

**Introduction:**

Tuberculosis (TB) is a chronic lung disease caused by *Mycobacterium tuberculosis*. Tobacco smoking and sharing of instruments have been reported to increase TB risk. In 2022, cannabis was legalized in Thailand. To address for the potential increase in cannabis use after legalization and the reported increased TB risk associated with cannabis usage, we aimed to estimate the odds ratio and population-attributable fraction (PAF) of different types of cannabis use.

**Materials and methods:**

A matched case–control study was conducted in the Songkhla Province of Southern Thailand in 2023. Face-to-face interviews were conducted to collect information on cannabis consumption. Multivariate logistic regression was performed to estimate the odds ratios representing TB risk from the independent variables. PAF was also calculated to compare the public health impacts of the variables.

**Results:**

Among the 148 TB cases and 117 healthy controls, we observed lower socioeconomic status and higher proportions of tobacco and alcohol use in these cases. Eleven percent of the controls were current cannabis users, while nearly 19% had ever experienced cannabis use. The proportions of ever-used, smoked, and shared cannabis use were significantly higher in these cases. After adjusting for covariates, the best-fit model showed an odds ratio of 4.22 (95% confidence interval: 1.47–12.07) for smoking and sharing a bong of cannabis. No statistical significance was found for the other types of consumption. PAF of smoked and shared bongs of cannabis was 12.16, which was slightly lower than that found in smoking tobacco (12.62).

**Conclusion:**

Increased numbers of cannabis users, especially shared smokers, may have an impact on TB risk in lower-middle-income countries, where TB is already highly prevalent.

## Introduction

Tuberculosis (TB) is a chronic lung disease caused by *Mycobacterium tuberculosis* (MTB). Currently, one-quarter of the world’s population is estimated to be infected with MTB (1). Both physical (2, 3) and mental burdens are well-established in patients with active pulmonary TB (4). Several risk factors have been identified to increase the risk of active TB, as demonstrated in previous studies. These factors include being male (5), older age (6, 7), poverty (8–10), and pre-existing HIV infection (11, 12). Increased TB risk from smoking tobacco is also well established (13–15); decreased immune response and impairment of immune cell and cilia functions have been suggested to mediate this increased risk (16). In addition to the immune-mediated risk, smoking tobacco through water pipes, or shisha, has been reported as an environmental TB risk due to the act of inhaling droplet containing MTB from contaminated mouthpieces during smoking session (17, 18).

Cannabis, also known as marijuana or gunja, is a flowering plant with psychoactive effects (19). According to the WHO, it is currently the most widely cultivated and consumed drug; moreover, approximately 2.5% of the global population is estimated to use cannabis (20). As a recreational drug, the popularity of cannabis lags behind that of caffeine, tobacco, and alcohol (19, 21). The collateral effects of the opioid epidemic have led to a gradual increase in interest in medicinal cannabis (22–24). Although their medicinal efficacy is not well established and several studies have reported increased cardiovascular risks from cannabis usage, such as heart failure and stroke (25, 26), cannabis and cannabinoid agents are currently used for various purposes, such as providing analgesia, alleviating cancer pain or neuropathic pain, and treating chemotherapy-induced nausea (27). Following a campaign by a political party to use cannabis to promote the domestic economy, both medicinal and recreational cannabis were legalized in Thailand in 2022 (28). A previous study conducted in US states reported an increase in the number of cannabis users after the legalization of recreational cannabis in four ethnicities including non-Hispanic black, Hispanic, non-Hispanic white and other. The increases in percentage of past-year cannabis use ranged from 1 to 3.7% (29). However, the impact of cannabis legalization in Thailand has not been well reported.

Several studies have reported mechanisms that could lead to increased TB risk from cannabis use, one of which is an increased inflammatory response. Compared to tobacco-only smokers, higher risks of prolonged coughing, chronic bronchitis, and chronic obstructive pulmonary disease (COPD) were found in co-users of tobacco and cannabis (30, 31). The increased inflammation from co-use could be due to the intake of both tobacco and cannabis smoke, which were reported to have similar physical and chemical properties (32). Another aspect similar to tobacco, cannabis can be smoked using various instruments. Following the smoking cannabis joint, the water pipe, bong, and hookah were among the most reported instruments used for cannabis consumption (33–35). Several studies have linked cannabis consumption through water pipes to pulmonary infections (36, 37). A necrotizing pneumonia case was found in individuals who inhaled contaminated pipe water (36). In addition, an increased TB risk from sharing cannabis water pipes with patients with TB was also reported in a contact tracing investigation in Queensland, Australia. In the same study, the odds ratio (OR) for acquiring TB infection through sharing cannabis water pipe was 2.22, following the highest OR of 4.91 in those who were household contacts of the TB case (37).

Although it has been shown that sharing bong among cannabis smokers could transmit TB (37, 38), no study at the population level has delineate this association, especially in lower-middle-income countries, such as Thailand, where TB and cannabis consumption are both highly prevalent. In 2021, the TB incidence in Thailand was predicted to be 143 per 100,000 individuals (39), while in the following year, a national poll regarding legalization of cannabis reported that 32% of participants ever consumed or used cannabis (40). This study aimed to conduct a case–control study to estimate the OR and population attributable fraction (PAF) of cannabis use and smoking, including sharing smoked cannabis, on active TB risk in our study population.

## Materials and methods

### Study setting

This study was conducted in Songkhla, southern Thailand, which is located on the coast of the Gulf of Thailand. The total population of the province is approximately 1.4 million people, with a population density of 194 per square kilometer (41). In Songkhla, the two largest hospitals responsible for TB treatment were Songklanagarind Hospital and Hatyai Hospital. The former, a university hospital, offers super-tertiary care, whereas the latter is classified as a regional hospital operated by the Ministry of Public Health. Thus, both hospitals were selected as the study hospitals. Regarding cannabis consumption, the Thailand National Poll reported a 32% prevalence of lifetime exposure to any type of cannabis. However, its prevalence at the provincial level has not been reported (40). Furthermore, this study was conducted in 2023, when both medicinal and recreational uses of cannabis were legal.

### Study design

A matched case–control study was conducted to compare cannabis usage and smoking between patients with active pulmonary TB and healthy controls in the same population.

### Case ascertainment

The case group was defined as active pulmonary TB patients. Patients were recruited from among outpatients diagnosed with active pulmonary TB based on bacteriological results or clinical diagnoses. Inclusion criteria comprised patients over 20 years old, residents of Songkhla Province, and those undergoing treatment at the study hospitals since January 1st, 2023. The latter criterion was placed to make sure that TB cases were diagnosed after cannabis was legalized in 2022. We excluded TB patients with diabetes mellitus, cancer, and HIV infection to prevent unnecessary heterogeneity in the exposure groups. The nursing teams at both study hospitals identified eligible cases for recruitment process. Information on the actual residential addresses of the patients who participated in the study was obtained for planning community control selection.

### Control selection

Controls in this study was matched with the cases using age group, sex, and community as the matching variables. Control matching varied between urban and rural cases due to differences in TB transmission, influenced by factors such as high population density, crowded living conditions, and low hygiene (42, 43). For rural areas, we defined a community as a village in which the patient resided. For the urban setting, we defined the study community as an alley of the patient’s home. The responsible healthcare center for each study community was approached. The study protocol was explained to the patients. The health offices were requested to invite health volunteers in charge to participate in the control selection. Information on the age group (10 year range) and sex of the patients in that community was used to match with the cases and guide the health volunteers to select controls. The exclusion criteria for controls were the same as those for the case group. Potential controls were not included if they had been diagnosed with TB. However, we did not attempt to exclude patients with TB from the control group using any tests, including chest radiography. When more than one eligible control was available for each TB case, volunteers were advised to randomly select one. Selected controls were then interviewed at a nearby community health center.

### Sample size calculation

This study sample size was calculated using two independent proportion formulas. For the exposure proportion in the control group, we used the previously reported prevalence of Thai people who had ever used cannabis for medicinal purposes, which was 0.32 for *P*_1_. We assumed the proportion of cannabis consumption among the cases (*P*_2_) to be 0.49, which was sufficient to generate an OR of 2. With a significance level of 0.05, a power of 0.8, and a 1:1 case-to-control ratio, the required total sample size was 148 cases and 148 controls.

### Data collection and variables

We started recruiting participants on March 9th, 2023, and ended data collection on December 5th, 2023. Both the case and control groups provided informed consent. Participants were informed that this was a survey on cannabis use without disclosing our hypothesis of the link to TB. Face-to-face interviews were conducted using a structured questionnaire. The questionnaire contained three sections including demographic information, tobacco and alcohol consumption, and cannabis consumption.

The outcome variable was active pulmonary TB status, as described in the aforementioned protocol. Our main exposure variables were cannabis exposure, including oral consumption and smoking status. Oral cannabis consumption included drinking cannabis tea, consuming foods enhanced or mixed with cannabis derivatives, and consuming commercial drinks containing cannabis. Smoking details included the form of preparation/instrument used for the smoking session, such as rolled joint, mixed cigarette, pipe, and bong. In this study, a bong was classified as any type of water pipe used for smoking cannabis. The social aspects of smoking included whether cannabis was shared during the session, such as sharing a cannabis joint or bong. As we hypothesized that cannabis sharing would be the main mechanism of TB transmission among cannabis users, we stratified smoking cannabis levels into non-smoking, smoking, and smoking and sharing cannabis separately for both smoking via a bong and rolled joints. Timing variables of consumption included age at start and quit (among ex-users). Frequencies and reasons for consumption were also collected. Other potential confounding variables included a history of tobacco smoking and alcohol consumption, education, occupation, and monthly income.

### Data management

During the interview process, data were collected directly and entered into a mobile REDCap application (44). The mobile data were transferred and securely stored in the REDCap database hosted by the Research and Development Office of Prince of Songkla University (45, 46). Data from REDCap were exported in a comma-separated value format (CSV), specifically for the analysis process, using the R program version 4.2.3 (47).

### Statistical analysis

Descriptive statistics were used to summarize all independent variables and stratified by the outcome variable. Frequency and percentage were used as categorical variables, whereas continuous variables were described using mean and standard deviation (SD) or median and interquartile range (IQR) based on their distribution. Univariate association analysis was conducted using either the chi-square test or Fisher’s exact test for small expected cell counts, while the Student’s t-test or Mann–Whitney U test was performed for continuous variables depending on the variable’s distribution. A logistic regression analysis was performed to adjust for the remaining confounding factors. The key independent variables maintained in the model were cannabis use and smoking. Other covariates included in the model as potential confounders were those that showed significantly association with the outcome in the univariate analysis (*p* < 0.2).

Because smoking tobacco is a strong confounder, a multivariate model was initially performed without it. This model was then compared with the model in which it was included. The coefficient/OR of cannabis in the model, including smoking tobacco, reflected its direct effect on TB risk. This stepwise modeling approach was used separately to analyze the effects of cannabis use and cannabis smoking. To compare the different models, we used the Akaike information criterion (AIC) values. The model with the lowest AIC was considered the best-fit model, given that the set of records was the same. In the case of collinearity between two exposure variables, the variable with the lowest AIC was selected. This model was then summarized to display the OR with a 95% confidence interval (CI) for each independent variable. In addition, the PAF based on Levin’s formula was computed for modifiable risk factors, particularly cannabis use (48). The significance level (*α*) for multivariate analysis was set at 0.05.

## Results

### Demographic characteristics

Of 152 eligible TB patients, 148 patients gave their consent, a response rate of 97.4%. We obtained 117 age-sex-community matched controls from the health volunteer recruitment. As these controls were readily recruited by the health workers, we did not have the information on the number of eligible subjects and the response rate. Table 1 shows the results of the univariate analysis. The patients in the case group were generally younger than the controls, had a lower education level, and had a higher proportion of unemployed individuals and office workers. Substance usage was more common in the case group, with a significantly higher proportion of individuals reporting ever-drank alcohol and tobacco, along with a higher prevalence of current smoker. Additionally, the monthly cigarette dosage was significantly higher in those who had smoked tobacco.

**Table 1:** Descriptive table stratified by case and control group status.

Variables	Control	Case	*p* value
Total	117	148	
Age			0.097^a^
Mean (SD)	55.1 (15.9)	51.8 (16.2)	
Age group			0.351^c^
≤ 40 years old	22 (18.8)	38 (25.7)	
41–60 years old	59 (50.4)	64 (43.2)	
> 60 years old	36 (30.8)	46 (31.1)	
Sex			0.492^c^
Male	71 (60.7)	97 (65.5)	
Female	46 (39.3)	51 (34.5)	
Education			0.042^c^
< High school	54 (46.2)	88 (59.5)	
≥ High school	63 (53.8)	60 (40.5)	
Occupation			0.065^c^
Business owner/freelance	31 (26.5)	22 (14.9)	
Laborer	32 (27.4)	40 (27)	
Office worker	12 (10.3)	26 (17.6)	
Retired/unemployed/student	42 (35.9)	60 (40.5)	
Monthly income (thousand baht) (*n* = 235)			0.388^b^
Median (IQR)	6 (0,15)	8 (0,15)	
Ever drank alcohol			0.004^c^
No	54 (46.2)	42 (28.4)	
Yes	63 (53.8)	106 (71.6)	
Current drinker			0.707^c^
No	78 (66.7)	103 (69.6)	
Yes	39 (33.3)	45 (30.4)	
Monthly drink count (*n* = 123)			0.004^b^
Median (IQR)	24 (10,60)	60 (16,120)	
Ever smoke			< 0.001^c^
No	69 (59)	49 (33.1)	
Yes	48 (41)	99 (66.9)	
Current smoker			0.035^c^
No	90 (76.9)	95 (64.2)	
Yes	27 (23.1)	53 (35.8)	
Monthly cigarette count (*n* = 137)			0.004^b^
Median (IQR)	155 (55,480)	300 (150,600)	

a Student’s *t*-test.

b Mann-Whitney U test.

c Chi-square test.

### Active pulmonary TB association with cannabis usage

The details of cannabis consumption among cases and controls are summarized in Table 2. Eleven percent of the controls were current cannabis users, whereas nearly 19% had used cannabis at least once before, most commonly through oral consumption (12.8%). Cannabis consumption among cases and controls was not significantly different among current users, quitting age, usage frequency, oral consumption, and health reasons behind cannabis use. Cases were more likely to have a history of ever using cannabis and smoking cannabis via a bong or joint, especially when sharing cannabis during the session. We obtained the ventilation status from 37 out of 38 bong smokers and 30 out of 33 joint smokers. Smoking in well-ventilated areas was reported by 33 (89.2%) of bong smokers and 26 (86.7%) of joint smokers.

**Table 2:** Comparison of cannabis use details among cases and controls.

Variables	Control	Case	*p* value
Total	117	148	
Current user (within 1 year)			0.303^b^
No	104 (88.9)	138 (93.2)	
Yes	13 (11.1)	10 (6.8)	
Ever used cannabis			0.002^b^
No	95 (81.2)	94 (63.5)	
Yes	22 (18.8)	54 (36.5)	
History of cannabis use			
Starting age (*n* = 76)			0.064^a^
Median (IQR)	22.5 (18.5,49.2)	19 (18,21)	
Year since last use (*n* = 49)			0.736^a^
Median (IQR)	15 (2,38)	10 (3,26.5)	
Frequency (*n* = 76)			0.366^b^
Occasionally	12 (54.5)	20 (37)	
Regularly	4 (18.2)	15 (27.8)	
Once or twice	6 (27.3)	19 (35.2)	
Usage via bong smoking			< 0.001^c^
No	111 (94.9)	116 (78.4)	
Smoke only	1 (0.9)	1 (0.7)	
Smoke and share	5 (4.3)	31 (20.9)	
Usage via joint smoking			0.005^b^
No	111 (94.9)	121 (81.8)	
Smoke only	3 (2.6)	11 (7.4)	
Smoke and share	3 (2.6)	16 (10.8)	
Usage via oral consumption			0.390^b^
No	102 (87.2)	135 (91.2)	
Yes	15 (12.8)	13 (8.8)	
Extra information among users			
Paid for cannabis (*n* = 51)			
No	15 (93.8)	20 (57.1)	
Yes	1 (6.2)	15 (42.9)	
Monthly spending in THB (*n* = 16)			
Median (IQR)	50	300 (100,450)	
Using cannabis for health reasons (*n* = 11)			
To increase appetite	0 (0)	4 (57.14)	
To treat insomnia	1 (25)	2 (28.57)	
To freshen up	0 (0)	1 (14.29)	
For unknown medicinal benefits	2 (50)	0 (0)	
Missing	1 (25)	0 (0)	

a Mann-Whitney U test.

b Chi-square test.

c Fisher’s exact test.

Table 3 compares the AICs of all prediction models for active pulmonary TB from various types of cannabis use with different combinations of covariates. Owing to the collinearity between smoking via the bong and joints, they were separately analyzed and displayed. Row A displays the results from ever-used cannabis, while rows B and C display those from smoking cannabis via bong and joint, respectively. Model 1, incorporating all exposure variables, showed a significant crude increased risk that remained significant after age, sex, education and occupation were included (Model 2). Of all the models, model 3B yielded the lowest AIC value and was therefore used to calculate the ORs and population-attributed fractions.

**Table 3:** Comparison of AIC values among predicting models for active pulmonary TB.

Key independent variables	Odds ratio		
	Model 1	Model 2	Model 3
Included covariates	–	Age group, sex, education, and occupation	Model 2 and history of tobacco smoking
(A)			
Ever used cannabis (ref = never)	2.48 (1.40,4.39)^*^	2.71 (1.47,5.01)^*^	2.00 (1.05,3.81)^*^
AIC	357.46	353.61	339.13
(B)			
Smoking cannabis via bong (ref = no)			
Smoke only	0.96 (0.06,15.49)	0.95 (0.06,16.23)	0.72 (0.04,12.38)
Smoke and shared	5.93 (2.23,15.80)^*^	6.35 (2.28,17.67)^*^	4.22 (1.47,12.07)^*^
AIC	351.36	349.76	336.78
(C)			
Smoking cannabis via joint (ref = no)			
Smoke only	3.36 (0.91,12.37)	4.50 (1.15,17.59)^*^	3.32 (0.84,13.22)
Smoke and shared	4.89 (1.39,17.24)^*^	5.45 (1.46,20.34)^*^	3.42 (0.89,13.05)
AIC	358.31	353.63	339.23

*Statistical significance at *p* < 0.05.

Table 4 displays the ORs (95% CI) of variables in model 3B. The effects from smoking and sharing cannabis via a bong were independently significant, as were the effects from tobacco smoking. The levels of ORs between smoking and sharing cannabis via a bong and tobacco smoking were similar (4.22; 95% CI: 1.47–12.07 and 4.36; 95% CI: 1.97–9.65, respectively). The percentages of PAF were 12.16 and 12.62 for the respective categories. Based on the likelihood ratio tests, all other independent variables in the model, except age, were also statistically significant.

**Table 4:** Odds ratios of active pulmonary TB.

Variable	OR (95% CI)	*P*_Wald’s test_	*P*_LR-test_	PAF (%)
Smoking cannabis via bong (ref = no)			0.011	
Smoke only	0.72 (0.04,12.38)	0.823		0
Smoke and shared	4.22 (1.47,12.07)	0.007		12.16
Age (ref = ≤ 40 years old)			0.169	
41–60 years old	0.51 (0.24,1.07)	0.077		
> 60 years old	0.5 (0.21,1.17)	0.109		
Sex (ref = male)			0.034	
Female	2.28 (1.04,5.04)	0.041		
Education (ref = ≥ High school)			0.023	
<High school	2.02 (1.1,3.72)	0.024		
Occupation (ref = business owner)			0.038	
Laborer	1.1 (0.47,2.55)	0.83		
Office worker	3.55 (1.36,9.27)	0.01		
Retired/unemployed/student	1.65 (0.75,3.61)	0.212		
Ever smoked tobacco (ref = no)	4.36 (1.97,9.65)	< 0.001	< 0.001	12.62

## Discussion

Our TB cases were more likely to have a lower SES than community-matched controls in terms of education. They were also more likely to consume alcohol and tobacco. Univariate analysis showed that both bong and joint sharing were associated with the disease. However, multivariate analysis suggested that bong sharing was a stronger risk factor, with an OR and PAF close to those of smoking tobacco.

Our study conformed with most previous research that found that low SES (49–51) and tobacco smoking were risk factors for TB (13). These factors are associated with addiction, including cannabis use, particularly in Thailand. Therefore, it is important to adjust for these factors when assessing the effects of cannabis in increasing pulmonary TB risk.

In this study, we tested the effects of different forms of cannabis. Oral consumption was not a risk factor, although it was the most common form of consumption. Leaves are commonly added to food to enhance its flavor and appetite. No plausible mechanism linking this to the risk of pulmonary TB was evident.

Without instrument sharing, we do not have enough evidence to suggest that cannabis smoking has the same effect as tobacco smoking. Notably, non-shared cannabis smoking is uncommon. This lack of evidence is likely explained by the small sample size of the non-sharing groups. Thus, we could not test the hypothesis that cannabis causes respiratory inflammation, which leads to increased TB risk.

In our setting, bong sharing was a significant risk factor for TB, which corresponds well with that reported in a previous contract-tracing study (37). MTB can remain viable in aerosol droplets for as long as 60 min (52). Bong sharing is a socializing activity among cannabis smokers. Aerosol droplets produced by a smoker can enter the bong cavity from the coughing of a patient with active pulmonary TB and be inhaled by the sharer shortly thereafter. Conversely, although more than 85% of bong and joint cannabis smokers were reported to smoke in well-ventilated areas, the possibility of inhaling aerosol droplets without sharing remains if the smokers are seated in close proximity.

Joint sharing is a common practice among cannabis smokers. Infectious MTB aerosol droplets can be transmitted to other smokers during a smoking session that is often conducted in a closed space (53). A similar mechanism has been reported for SARS-CoV-2 transmission among tobacco smokers who share the same cigarette (54). The collinearity between joint and bong sharing compelled us to opt supporting only one interest. Our choice to choose bong sharing as the main interest was due to its significant adjusted ORs (4.22; 95% CI: 1.47–12.07), compared to the non-significant OR of joint sharing (3.42; 95% CI: 0.89–13.05). Our study lacked sufficient power to demonstrate the effect of joint sharing because of the small sample size of joint sharing behavior.

Tobacco smoking had similar levels of adjusted OR and PAF as those calculated from the sharing of bongs of cannabis. Tobacco smoking causes ciliary dysfunction and reduces local immunity against MTB infection (55). Although sharing instruments for tobacco smoking increases the risk of TB transmission (56), this behavior is not as common as that observed in cannabis smokers. Therefore, at the population level, the mechanisms attributed to TB cases are different.

The strengths of this study lie in the time and place where the study was conceptualized. Thailand, a high TB burden country, and also the first country in Asia to legalize cannabis. It added evidence of this association in general population setting from those derived from a TB contact tracing study (37).

A limitation of the study might come from uncommon setting that number of cases was higher than number of controls, as not all cases could have an eligible control to match with. However, the post-hoc analysis confirmed sufficient power for our hypothesis testing. In addition, sensitivity analyses using only cases with matched control (Supplementary Tables 1–3) showed similar results in both univariate and multivariate analyses, with marginal differences in proportions in the former and a slightly lower odds ratio from sharing bong of cannabis in the latter. These results suggest that not having enough controls for a 1:1 case–control ratio could slightly overestimate the association, as the incomplete control group was not a perfect representation of the matched-population. However, including cases without matched control in the analysis provided slightly higher statistical power. The collinearity between joint and bong sharing among cannabis smokers disabled us to separate these two factors well enough. Our TB cases (and thus also the matched control) were generally older than cannabis consumers in the general population. The prevalence of tobacco smoking in the control group (23.1%) was much higher than in the general population (19%), as reported in a 2017 national survey (57). This might be due to the fact that our study subjects resided in low socio-economic areas. In addition, we excluded TB patients with immunocompromised conditions. This may reduce the generalizability of our findings, as it may not reflect the general TB patient population, especially for PAF. Moreover, the time lag between policy change in 2022 and the study period in 2023 was just 1 year. The association found may not be due to the change in the policy. Therefore, the findings of this study must be interpreted with caution.

Most cannabis smokers have started smoking since they were young. Recent changes in the prevalence of cannabis smoking were not observed in the present study. However, the legalization of cannabis in other countries has been followed by an increase in the number of users (29). Therefore, this policy may have an impact on TB risk in lower-middle-income countries where TB is already highly prevalent.

## Data Availability

The original contributions presented in the study are included in the Supplementary material, further inquiries can be directed to the corresponding author.

## References

[1] Geneva: World Health Organization. Global tuberculosis reports. Geneva, Switzerland: WHO (2021).

[2] Ravimohan S Kornfeld H Weissman D Bisson GP. Tuberculosis and lung damage: from epidemiology to pathophysiology. Eur Respir Rev. (2018) 27:170077. doi: 10.1183/16000617.0077-2017 29491034 PMC6019552

[3] Allwood BW van der Zalm MM Amaral AFS Byrne A Datta S Egere U. Post-tuberculosis lung health: perspectives from the first international symposium. Int J Tuberc Lung Dis. (2020) 24:820–8. doi: 10.5588/ijtld.20.0067, PMID: 32912387

[4] Wang X Li XL Zhang Q Zhang J Chen HY Xu WY. A survey of anxiety and depressive symptoms in pulmonary tuberculosis patients with and without tracheobronchial tuberculosis. Front Psychol. (2018) 9:308. doi: 10.3389/fpsyt.2018.00308, PMID: 30072924 PMC6060437

[5] Horton KC MacPherson P Houben RMGJ White RG Corbett EL. Sex differences in tuberculosis burden and notifications in low- and middle-income countries: a systematic review and Meta-analysis. PLoS Med. (2016) 13:e1002119. doi: 10.1371/journal.pmed.1002119, PMID: 27598345 PMC5012571

[6] Manabe T Takasaki J Kudo K. Seasonality of newly notified pulmonary tuberculosis in Japan, 2007–2015. BMC Infect Dis. (2019) 19:497. doi: 10.1186/s12879-019-3957-8, PMID: 31170932 PMC6555020

[7] Li S Li YF Song WM Zhang QY Liu SQ Xu TT. Population aging and trends of pulmonary tuberculosis incidence in the elderly. BMC Infect Dis. (2021) 21:302. doi: 10.1186/s12879-021-05994-z, PMID: 33765943 PMC7993467

[8] Hawker JI Bakhshi SS Ali S Farrington CP. Ecological analysis of ethnic differences in relation between tuberculosis and poverty. BMJ. (1999) 319:1031–4. doi: 10.1136/bmj.319.7216.1031, PMID: 10521193 PMC28253

[9] Long ER. Constitution and related factors in resistance to tuberculosis. Arch Pathol. (1941) 32:122–62.

[10] Cantwell MF McKenna MT McCray E Onorato IM. Tuberculosis and race/ethnicity in the United States: impact of socioeconomic status. Am J Respir Crit Care Med. (1998) 157:1016–20. doi: 10.1164/ajrccm.157.4.9704036 9563713

[11] Corbett EL Watt CJ Walker N Maher D Williams BG Raviglione MC. The growing burden of tuberculosis: global trends and interactions with the HIV epidemic. Arch Intern Med. (2003) 163:1009–21. doi: 10.1001/archinte.163.9.1009 12742798

[12] Sharma SK Mohan A Kadhiravan T. HIV-TB co-infection: epidemiology, diagnosis & management. Indian J Med Res. (2005) 121:550–67. PMID: 15817963

[13] Bates MN Khalakdina A Pai M Chang L Lessa F Smith KR. Risk of tuberculosis from exposure to tobacco smoke: a systematic review and Meta-analysis. Arch Intern Med. (2007) 167:335–42. doi: 10.1001/archinte.167.4.335 17325294

[14] World Health Organization. Smoking and tuberculosis: a dangerous combination. (2018). Available from: https://www.who.int/europe/news/item/22-03-2018-smoking-and-tuberculosis-a-dangerous-combination (Accessed December 24, 2023).

[15] Alavi-Naini R Sharifi-Mood B Metanat M. Association between tuberculosis and smoking. Int J High Risk Behav Addict. (2012) 1:71–4. doi: 10.5812/ijhrba.5215, PMID: 24971236 PMC4070106

[16] Arcavi L Benowitz NL. Cigarette smoking and infection. Arch Intern Med. (2004) 164:2206–16. doi: 10.1001/archinte.164.20.2206 15534156

[17] Kamel M Elyes H Sophia B Raya S Abdellatif C. Pulmonary tuberculosis in narghile (Ng) lighters. Eur Respir J. (2002) 20:555s. Available at: https://onlinelibrary.wiley.com/doi/pdf/10.1100/tsw.2006.332

[18] Urkin J Ochaion R Peleg A. Hubble bubble equals trouble: the hazards of water pipe smoking. Sci World J. (2006) 6:1990–7. doi: 10.1100/tsw.2006.332, PMID: 17369998 PMC5917204

[19] Lafaye G Karila L Blecha L Benyamina A. Cannabis, cannabinoids, and health. Dialogues Clin Neurosci. (2017) 19:309–16. doi: 10.31887/DCNS.2017.19.3/glafaye, PMID: 29302228 PMC5741114

[20] World Health Organization. Alcohol, drugs and addictive Behaviours unit: Cannabis. (2023). Available from: https://www.who.int/teams/mental-health-and-substance-use/alcohol-drugs-and-addictive-behaviours/drugs-psychoactive/cannabis (Accessed December 24, 2023).

[21] ElSohly MA. Marijuana and the cannabinoids. US: Springer Science & Business Media (2007). 323 p.

[22] Mahabir VK Merchant JJ Smith C Garibaldi A. Medical cannabis use in the United States: a retrospective database study. J Cannabis Res. (2020) 2:32. doi: 10.1186/s42238-020-00038-w, PMID: 33526110 PMC7819290

[23] Pratt M Stevens A Thuku M Butler C Skidmore B Wieland LS. Benefits and harms of medical cannabis: a scoping review of systematic reviews. Syst Rev. (2019) 8:320. doi: 10.1186/s13643-019-1243-x, PMID: 31823819 PMC6905063

[24] Wang Y Robinson KR Fechtel H Hartog A. Medical Cannabis use and its impact on health among older adults: recent research findings and future directions. Curr Addict Rep. (2023) 10:837–43. doi: 10.1007/s40429-023-00519-x 38586531 PMC10997349

[25] Jeffers AM Glantz S Byers AL Keyhani S. Association of Cannabis use with Cardiovascular Outcomes among US adults. J Am Heart Assoc. (2024) 13:e030178. doi: 10.1161/JAHA.123.030178, PMID: 38415581 PMC10944074

[26] Page RL Allen LA Kloner RA Carriker CR Martel C Morris AA. Medical marijuana, recreational Cannabis, and cardiovascular health: a scientific statement from the American Heart Association. Circulation. (2020) 142:e131–52. doi: 10.1161/CIR.0000000000000883 32752884

[27] Aguilar S Gutiérrez V Sanchez LM Nougier M. Medicinal cannabis policies and practices around the world. (2018). Available from: https://www.semanticscholar.org/paper/Medicinal-cannabis-policies-and-practices-around-Aguilar-Guti%C3%A9rrez/973596c856fa48190ac33a54e4999fbc4cfd7b65 (Accessed December 24, 2023).

[28] Government of Thailand. Thailand narcotics act. Thailand: Government of Thailand (2019).

[29] Martins SS Segura LE Levy NS Mauro PM Mauro CM Philbin MM. Racial and ethnic differences in Cannabis use following legalization in US states with medical Cannabis Laws. JAMA Netw Open. (2021) 4:e2127002. doi: 10.1001/jamanetworkopen.2021.27002, PMID: 34570205 PMC8477268

[30] Moore BA Augustson EM Moser RP Budney AJ. Respiratory effects of marijuana and tobacco use in a U.S. Sample J Gen Intern Med. (2005) 20:33–7. doi: 10.1111/j.1525-1497.2004.40081.x, PMID: 15693925 PMC1490047

[31] Tan WC Lo C Jong A Xing L FitzGerald MJ Vollmer WM. Marijuana and chronic obstructive lung disease: a population-based study. CMAJ. (2009) 180:814–20. doi: 10.1503/cmaj.081040, PMID: 19364790 PMC2665947

[32] Graves BM Johnson TJ Nishida RT Dias RP Savareear B Harynuk JJ. Comprehensive characterization of mainstream marijuana and tobacco smoke. Sci Rep. (2020) 10:7160. doi: 10.1038/s41598-020-63120-6, PMID: 32345986 PMC7188852

[33] Singh T Kennedy SM Sharapova SS Schauer GL Rolle IV. Modes of ever marijuana use among adult tobacco users and non-tobacco users—styles 2014. J Subst Use. (2016) 21:631–5. doi: 10.3109/14659891.2015.1122100, PMID: 27840591 PMC5103614

[34] Baggio S Deline S Studer J Mohler-Kuo M Daeppen JB Gmel G. Routes of administration of cannabis used for nonmedical purposes and associations with patterns of drug use. J Adolesc Health. (2014) 54:235–40. doi: 10.1016/j.jadohealth.2013.08.013, PMID: 24119417

[35] Domínguez-Salas S Piqueras-Torrico M Allande-Cussó R Gómez-Salgado J Andrés-Villas M. The use of water pipe and its impact on university students’ lifestyle and their psychological distress: a cross-sectional study. Rev Esp Salud Publica. (2020) 94:e202012156 33319772 PMC11582827

[36] Kumar AN Soo CI Ng BH Hassan T Ban AY Manap RA. Marijuana “bong” pseudomonas lung infection: a detrimental recreational experience. Respirol Case Rep. (2017) 6:e00293. doi: 10.1002/rcr2.293 29321937 PMC5756717

[37] Munckhof WJ Konstantinos A Wamsley M Mortlock M Gilpin C. A cluster of tuberculosis associated with use of a marijuana water pipe. Int J Tuberc Lung Dis. (2003) 7:860–5. PMID: 12971670

[38] Thu K Hayes M Miles S Tierney L Foy A. Marijuana ‘bong’ smoking and tuberculosis. Intern Med J. (2013) 43:456–8. doi: 10.1111/imj.12089, PMID: 23551310

[39] Division of Tuberculosis. Thailand operational plan to end tuberculosis, phase 2 (2023–2027). Thailand: Department of Disease Control, Ministry of Public Health Thailand (2023).

[40] National Institute of Development Administration. NIDA poll: unlocking marijuana from a drug account. (2022). Available from: https://nidapoll.nida.ac.th/survey_detail?survey_id=579 (Accessed February 4, 2023).

[41] The Bureau of Registration Administration. Statistics, population and house statistics for the year 2023. Thailand: Department of Provincial Administration (2023).

[42] Mutembo S Mutanga JN Musokotwane K Kanene C Dobbin K Yao X. Urban-rural disparities in treatment outcomes among recurrent TB cases in Southern Province, Zambia. BMC Infect Dis. (2019) 19:1087. doi: 10.1186/s12879-019-4709-5, PMID: 31888518 PMC6938018

[43] Noykhovich E Mookherji S Roess A. The risk of tuberculosis among populations living in slum settings: a systematic review and Meta-analysis. J Urban Health. (2019) 96:262–75. doi: 10.1007/s11524-018-0319-6, PMID: 30341562 PMC6458189

[44] Harris PA Delacqua G Taylor R Pearson S Fernandez M Duda SN. The REDCap Mobile application: a data collection platform for research in regions or situations with internet scarcity. J Am Med Inform Assoc. (2021) 4, 1–7. doi: 10.1093/jamiaopen/ooab078, PMID: 34527889 PMC8435658

[45] Harris PA Taylor R Thielke R Payne J Gonzalez N Conde JG. Research electronic data capture (REDCap) - a metadata-driven methodology and workflow process for providing translational research informatics support. J Biomed Inform. (2009) 42:377–81. doi: 10.1016/j.jbi.2008.08.010, PMID: 18929686 PMC2700030

[46] Harris PA Taylor R Minor BL Elliott V Fernandez M O’Neal L. The REDCap consortium: building an international community of software platform partners. J Biomed Inform. (2019) 95:103208. doi: 10.1016/j.jbi.2019.103208, PMID: 31078660 PMC7254481

[47] R Core Team. R: A language and environment for statistical computing. Vienna, Austria: R Foundation for Statistical Computing (2023).

[48] Levin ML. The occurrence of lung cancer in man. Acta Unio Int Contra Cancrum. (1953) 9:531–41. PMID: 13124110

[49] Olson NA Davidow AL Winston CA Chen MP Gazmararian JA Katz DJ. A national study of socioeconomic status and tuberculosis rates by country of birth, United States, 1996–2005. BMC Public Health. (2012) 12:365. doi: 10.1186/1471-2458-12-365, PMID: 22607324 PMC3506526

[50] Choi SW Im JJ Yoon SE Kim SH Cho JH Jeong SJ. Lower socioeconomic status associated with higher tuberculosis rate in South Korea. BMC Pulm Med. (2023) 23:418. doi: 10.1186/s12890-023-02713-z, PMID: 37907868 PMC10619321

[51] Nguipdop-Djomo P Rodrigues LC Smith PG Abubakar I Mangtani P. Drug misuse, tobacco smoking, alcohol and other social determinants of tuberculosis in UK-born adults in England: a community-based case-control study. Sci Rep. (2020) 10:5639. doi: 10.1038/s41598-020-62667-8, PMID: 32221405 PMC7101386

[52] Lever MS Williams A Bennett AM. Survival of mycobacterial species in aerosols generated from artificial saliva. Lett Appl Microbiol. (2000) 31:238–41. doi: 10.1046/j.1365-2672.2000.00807.x, PMID: 10972736

[53] Berg CJ Buller DB Schauer GL Windle M Stratton E Kegler MC. Rules regarding marijuana and its use in personal residences: findings from marijuana users and nonusers recruited through social media. J Environ Public Health. (2015) 2015:476017:1–7. doi: 10.1155/2015/476017 26576162 PMC4630400

[54] Yasri S Wiwanitkit V. Sharing cigarette smoking and COVID-19 outbreak in a party group. Int J Prev Med. (2020) 11:50. doi: 10.4103/ijpvm.IJPVM_121_20, PMID: 32577180 PMC7297418

[55] van Zyl Smit RN Pai M Yew WW Leung CC Zumla A Bateman ED. Global lung health: the colliding epidemics of tuberculosis, tobacco smoking. HIV and COPD Eur Respir J. (2010) 35:27–33. doi: 10.1183/09031936.00072909, PMID: 20044459 PMC5454527

[56] Marchetti AU Boss OL Schenker CM Kälin K. Water-pipe smoking as a risk factor for transmitting *Mycobacterium tuberculosis*. Eur J Case Rep Intern Med. (2019) 7:001342. doi: 10.12890/2019_001342 32015968 PMC6993912

[57] National Statistical Office. The smoking and drinking behavior survey. Bangkok, Thailand: National Statistical Office (2017).

